# Supplementary material for: Genome-wide association study reveals a locus in ADARB2 for complete freedom from headache in Danish Blood Donors
Source: Commun Biol. 2024 May 27;7:646. doi: 10.1038/s42003-024-06299-y (PMC11130207; doi:10.1038/s42003-024-06299-y)
Supplement: Supplementary file 2 — Supplementary Material [file 42003_2024_6299_MOESM2_ESM.pdf]

Supplementary Table 1: Age of cases and controls in the discovery and replication cohorts.

	Discovery cohort		Replication cohort	
	Cases	Controls	Cases	Controls
Age for women, mean (SD)	50.5 (15.4)	45.1 (14.0)	41.1 (14.6)	38.6 (13.2)
Age for men, mean (SD)	52.5 (14.7)	47.1 (13.8)	45.8 (13.8)	40.3 (12.7)

Age: age in years. SD: standard deviation.

Supplementary Figure 1: Scree plot of variance in the discovery cohort explained by principal component 1 to 10.

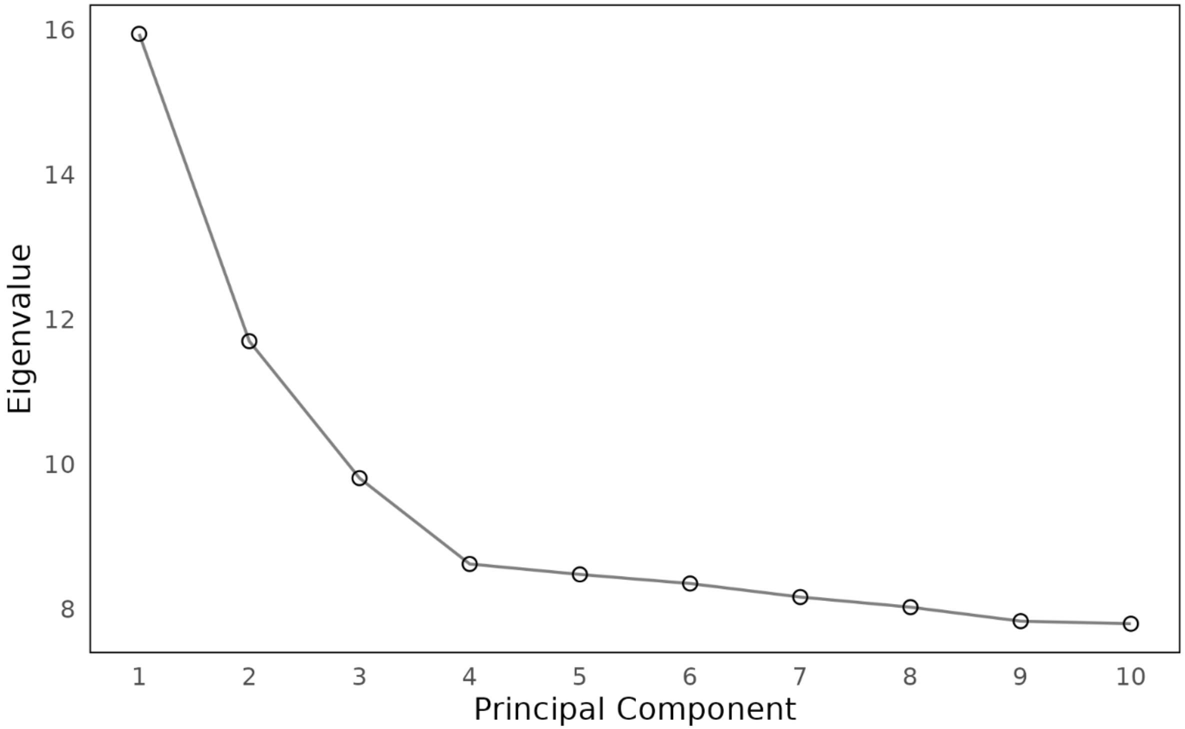

Supplementary Table 2:
International Headache Genetics Consoritum, IHGC

Name	Affiliation 1	Affiliation 2	Affiliation 3	Affiliation 4
Verneri Anttila	Analytical and Translational Genetics Unit, Department of Medicine, Massachusetts General Hospital and Harvard Medical School, Boston, Massachusetts, USA.	Program in Medical and Population Genetics, Broad Institute of MIT and Harvard, Cambridge, Massachusetts, USA.	Stanley Center for Psychiatric Research, Broad Institute of MIT and Harvard, Cambridge, Massachusetts, USA.	
Ville Artto	Department of Neurology, Helsinki University Central Hospital, Helsinki, Finland.			
Andrea C Belin	Department of Neuroscience, Karolinska Institutet, Stockholm, Sweden.			
Anna Bjornsdottir	Neurology private practice, Laeknasetrid, Reykjavik, Iceland.			
Gyda Bjornsdottir	deCODE genetics/Amgen Inc., Reykjavik, Iceland.			
Dorret I Boomsma	Netherlands Twin Register, Department of Biological Psychology, Vrije Universiteit, Amsterdam, the Netherlands.			
Sigrid Børte	K.G. Jebsen Center for Genetic Epidemiology, Department of Public Health and Nursing, Faculty of Medicine and Health Sciences, Norwegian University of Science and Technology, Trondheim, Norway.	Institute of Clinical Medicine, Faculty of Medicine, University of Oslo, Oslo, Norway.	Research and Communication Unit for Musculoskeletal Health, Department of Research, Innovation and Education, Division of Clinical Neuroscience, Oslo University Hospital, Oslo, Norway.	
Mona A Chalmer	Danish Headache Center, Department of Neurology, Copenhagen University Hospital, Copenhagen, Denmark.			
Daniel I Chasman	Department of Medicine, Division of Preventive Medicine, Brigham and Women's Hospital, Boston, Massachusetts, USA.	Harvard Medical School, Boston, Massachusetts, USA.		

Bru Cormand	Department of Genetics, Spain Centre for Biomedical Network Research on Rare Diseases, University of Barcelona, Barcelona, Spain.	
Ester Cuenca-Leon	Pediatric Neurology Research Group, Vall d'Hebron Research Institute, Barcelona, Spain.	
George Davey-Smith	University of Bristol/Medical Research Council Integrative Epidemiology Unit, University of Bristol, Bristol, UK.	
Irene de Boer	Department of Neurology, Leiden University Medical Centre, Leiden, the Netherlands.	
Martin Dichgans	Institute for Stroke and Dementia Research, University Hospital, LMU Munich, Munich, Germany.	Munich Cluster for Systems Neurology, Munich, Germany.
Tonu Esko	Estonian Biobank Registry, the Estonian Genome Center, University of Tartu, Tartu, Estonia.	
Tobias Freilinger	Department of Neurology, Klinikum Passau, Passau, Germany.	Department of Neurology and Epileptology, Hertie Institute for Clinical Brain Research, University of Tuebingen, Tuebingen, Germany.
Padhraig Gormley	GSK Inc., Cambridge, Massachusetts, USA.	
Lyn R Griffiths	Centre for Genomics and Personalised Health, Queensland University of Technology, Brisbane, Queensland, Australia.	
Eija Hämäläinen	Institute for Molecular Medicine Finland, Helsinki Institute of Life Science, University of Helsinki, Helsinki, Finland.	
Thomas F Hansen	Danish Headache Center, Department of Neurology, Copenhagen University Hospital, Copenhagen, Denmark.	Novo Nordic Foundation Center for Protein Research, Copenhagen University, Copenhagen, Denmark.
Aster VE Harder	Department of Neurology, Leiden University Medical Centre, Leiden, the Netherlands.	Department of Human Genetics, Leiden University Medical Centre, Leiden, the Netherlands.
Heidi Hautakangas	Institute for Molecular Medicine Finland, Helsinki Institute of Life Science, University of Helsinki, Helsinki, Finland.	

Marjo Hiekkala	Folkhälsan Research Center, Helsinki, Finland.			
Maria G Hrafnsdottir	Landspitali University Hospital, Reykjavik, Iceland.			
M. Arfan Ikram	Department of Epidemiology, Erasmus University Medical Center, Rotterdam, the Netherlands.			
Marjo-Riitta Järvelin	Department of Epidemiology and Biostatistics, MRC-PHE Centre for Environment and Health, School of Public Health, Imperial College London, London, UK.	Center for Life Course Health Research, Faculty of Medicine, University of Oulu, Oulu, Finland.	Unit of Primary Health Care, Oulu University Hospital, OYS, Oulu, Finland.	Department of Life Sciences, College of Health and Life Sciences, Brunel University London, London, UK.
Risto Kajanne	Institute for Molecular Medicine Finland, Helsinki Institute of Life Science, University of Helsinki, Helsinki, Finland.			
Mikko Kallela	Department of Neurology, Helsinki University Central Hospital, Helsinki, Finland.			
Jaakko Kaprio	Institute for Molecular Medicine Finland, Helsinki Institute of Life Science, University of Helsinki, Helsinki, Finland.			
Mari Kaunisto	Folkhälsan Research Center, Helsinki, Finland.			
Lisette JA Kogelman	Danish Headache Center, Department of Neurology, Copenhagen University Hospital, Copenhagen, Denmark.			
Espen S Kristoffersen	Research and Communication Unit for Musculoskeletal Health, Department of Research, Innovation and Education, Division of Clinical Neuroscience, Akershus University Hospital and University of Oslo, Oslo, Norway.	Department of General Practice, Institute of Health and Society, University of Oslo, Oslo, Norway.	Department of Neurology, Akershus University Hospital, Lørenskog, Norway.	
Christian Kubisch	Institute of Human Genetics, University Medical Center Hamburg-Eppendorf, Hamburg, Germany.			
Mitja Kurki	Psychiatric and Neurodevelopmental Genetics Unit, Department of Medicine, Massachusetts General Hospital, Boston, Massachusetts, USA.			
Tobias Kurth	Institute of Public Health, Charité – Universitätsmedizin, Berlin.			
Lenore Launer	Laboratory of Epidemiology and Population Sciences, Intramural Research Program, National Institute on Aging, Bethesda, Maryland, USA.			
Terho Lehtimäki	Department of Clinical Chemistry, Fimlab Laboratories, and Finnish Cardiovascular Research Center - Tampere, Faculty of Medicine			

	and Health Technology, Tampere University, Tampere, Finland.		
Davor Lessel	Institute of Human Genetics, University Medical Center Hamburg-Eppendorf, Hamburg, Germany.		
Lannie Ligthart	Netherlands Twin Register, Department of Biological Psychology, Vrije Universiteit, Amsterdam, the Netherlands.		
Sigurdur H Magnusson	deCODE genetics/Amgen Inc., Reykjavik, Iceland.		
Rainer Malik	Institute for Stroke and Dementia Research, University Hospital, LMU Munich, Munich, Germany.		
Bertram Müller-Myhsok	Max Planck Institute of Psychiatry, Munich, Germany.		
Carrie Northover	23&Me Inc., Mountain View, California, USA.		
Dale R Nyholt	School of Biomedical Sciences, Faculty of Health, Centre for Genomics and Personalised Health, Centre for Data Science, Queensland University of Technology, Brisbane, Queensland, Australia.		
Jes Olesen	Danish Headache Center, Department of Neurology, Copenhagen University Hospital, Copenhagen, Denmark.		
Aarno Palotie	Institute for Molecular Medicine Finland, Helsinki Institute of Life Science, University of Helsinki, Helsinki, Finland.	University of Helsinki, Helsinki, Finland.	
Priit Palta	Institute for Molecular Medicine Finland, Helsinki Institute of Life Science, University of Helsinki, Helsinki, Finland.		
Linda M Pedersen	Department of Research, Innovation and Education, Division of Clinical Neuroscience, Oslo University Hospital, Oslo, Norway.		
Nancy Pedersen	Department of Medical Epidemiology and Biostatistics, Karolinska Institutet, Stockholm, Sweden.		
Matti Pirinen	Institute for Molecular Medicine Finland, Helsinki Institute of Life Science, University of Helsinki, Helsinki, Finland.	Department of Mathematics and Statistics, University of Helsinki, Helsinki, Finland.	Department of Public Health, University of Helsinki, Helsinki, Finland.
Danielle Posthuma	Department of Complex Trait Genetics, Center for Neurogenomics and Cognitive Research,		

	Neuroscience Campus Amsterdam, VU University, Amsterdam, The Netherlands.			
Patricia Pozo-Rosich	Headache Unit, Neurology Department, Vall d'Hebron University Hospital, Barcelona, Spain.			
Alice Pressman	Sutter Health, Sacramento, California, USA.			
Olli Raitakari	Centre for Population Health Research, University of Turku, Turku University Hospital, Turku, Finland.	Research Centre of Applied and Preventive Cardiovascular Medicine, University of Turku, Turku, Finland.	Department of Clinical Physiology and Nuclear Medicine, Turku University Hospital, Turku, Finland.	
Caroline Ran	Department of Neuroscience, Karolinska Institutet, Stockholm, Sweden.			
Gudrun R Sigurdardottir	Neurology private practice, Laeknasetrid, Reykjavik, Iceland.			
Hreinn Stefansson	deCODE genetics/Amgen Inc., Reykjavik, Iceland.			
Kari Stefansson	deCODE genetics/Amgen Inc., Reykjavik, Iceland.			
Olafur A Sveinsson	Landspitali University Hospital, Reykjavik, Iceland.			
Gisela M Terwindt	Department of Neurology, Leiden University Medical Centre, Leiden, the Netherlands.			
Thorgeir E Thorgeirsson	deCODE genetics/Amgen Inc., Reykjavik, Iceland.			
Arn MJM van den Maagdenberg	Department of Neurology, Leiden University Medical Centre, Leiden, the Netherlands.	Department of Human Genetics, Leiden University Medical Centre, Leiden, the Netherlands.		
Cornelia van Duijn	Department of Epidemiology, Erasmus University Medical Centre, Rotterdam, the Netherlands.			
Maija Wessman	Institute for Molecular Medicine Finland, Helsinki Institute of Life Science, University of Helsinki, Helsinki, Finland.	Folkhälsan Research Center, Helsinki, Finland.		
Bendik S Winsvold	K.G. Jebsen Center for Genetic Epidemiology, Department of Public Health and Nursing, Faculty of Medicine and Health Sciences, Norwegian University of Science and Technology, Trondheim, Norway.	Department of Research, Innovation and Education, Division of Clinical Neuroscience, Oslo University Hospital, Oslo, Norway.	Department of Neurology, Oslo University Hospital, Oslo, Norway.	

John-Anker Zwart	K.G. Jebsen Center for Genetic Epidemiology, Department of Public Health and Nursing, Faculty of Medicine and Health Sciences, Norwegian University of Science and Technology, Trondheim, Norway.	Institute of Clinical Medicine, Faculty of Medicine, University of Oslo, Oslo, Norway.	Department of Research, Innovation and Education, Division of Clinical Neuroscience, Oslo University Hospital, Oslo, Norway.

Danish Blood Donor Study Genomic Consortia:

First name	Surname	Affiliation 1	Affiliation 2
Karina	Banasik	Novo Nordisk Foundation Center for Protein Research, Faculty of Health and Medical Sciences, University of Copenhagen, Copenhagen, Denmark	
Jakob	Bay	Department of Clinical Immunology, Zealand University Hospital, Køge, Denmark	
Jens Kjærgaard	Boldsen	Department of Clinical Immunology, Aarhus University Hospital, Aarhus, Denmark	
Thorsten	Brodersen	Department of Clinical Immunology, Zealand University Hospital, Køge, Denmark	
Søren	Brunak	Novo Nordisk Foundation Center for Protein Research, Faculty of Health and Medical Sciences, University of Copenhagen, Copenhagen, Denmark	
Alfonso	Buil Demur	Institute of Biological Psychiatry, Mental Health Centre, Sct. Hans, Copenhagen University Hospital, Roskilde, Denmark	
Lea Arregui Nordahl	Christoffersen	Department of Clinical Immunology, Zealand University Hospital, Køge, Denmark	
Maria	Didriksen	Department of Clinical Immunology, Copenhagen University Hospital, Rigshospitalet, Copenhagen, Denmark	
Khoa Manh	Dinh	Department of Clinical Immunology, Aarhus University Hospital, Aarhus, Denmark	
Joseph	Dowsett	Department of Clinical Immunology, Copenhagen University Hospital, Rigshospitalet, Copenhagen, Denmark	
Christian	Erikstrup	Department of Clinical Immunology, Aarhus University Hospital, Aarhus, Denmark	Department of Clinical Medicine, Health, Aarhus University, Aarhus, Denmark

Bjarke	Feenstra	Department of Clinical Immunology, Copenhagen University Hospital, Rigshospitalet, Copenhagen, Denmark	Department of Epidemiology Research, Statens Serum Institut, Copenhagen, Denmark
Frank	Geller	Department of Clinical Immunology, Copenhagen University Hospital, Rigshospitalet, Copenhagen, Denmark	Department of Epidemiology Research, Statens Serum Institut, Copenhagen, Denmark
Daniel	Gudbjartsson	deCODE Genetics, Reykjavik, Iceland	
Thomas Folkmann	Hansen	Danish Headache Center, Department of Neurology, Copenhagen University Hospital, Rigshospitalet-Glostrup, Copenhagen, Denmark	
Dorte	Helenius Mikkelsen	Institute of Biological Psychiatry, Mental Health Centre, Sct. Hans, Copenhagen University Hospital, Roskilde, Denmark	
Lotte	Hindhede	Department of Clinical Immunology, Aarhus University Hospital, Aarhus, Denmark	
Henrik	Hjalgrim	Danish Cancer Society Research Center, Copenhagen, Denmark	Department of Epidemiology Research, Statens Serum Institut, Copenhagen, Denmark
Jakob	Hjorth von Stemann	Department of Clinical Immunology, Copenhagen University Hospital, Rigshospitalet, Copenhagen, Denmark	
Bitten Aagaard	Jensen	Department of Clinical Immunology, Aalborg University Hospital, Aalborg, Denmark	
Andrew	Joseph Schork	Institute of Biological Psychiatry, Mental Health Centre, Sct. Hans, Copenhagen University Hospital, Roskilde, Denmark	
Kathrine	Kaspersen	Department of Clinical Immunology, Aarhus University Hospital, Aarhus, Denmark	
Bertram Dalskov	Kjerulff	Department of Clinical Immunology, Aarhus University Hospital, Aarhus, Denmark	
Mette	Kongstad	Department of Clinical Immunology, Copenhagen University Hospital, Rigshospitalet, Copenhagen, Denmark	
Susan	Mikkelsen	Department of Clinical Immunology, Aarhus University Hospital, Aarhus, Denmark	
Christina	Mikkelsen	Department of Clinical Immunology, Copenhagen University Hospital, Rigshospitalet, Copenhagen, Denmark	

Ioanna	Nissen	Department of Clinical Immunology, Copenhagen University Hospital, Rigshospitalet, Copenhagen, Denmark	
Mette	Nyegaard	Department of Health Science and Technology, Faculty of Medicine, Aalborg Univeristy, Aalborg, Denmark	
Sisse Rye	Ostrowski	Department of Clinical Immunology, Copenhagen University Hospital, Rigshospitalet, Copenhagen, Denmark	Department of Clinical Medicine, Faculty of Health and Medical Sciences, University of Copenhagen, Copenhagen, Denmark
Ole Birger	Pedersen	Department of Clinical Immunology, Zealand University Hospital, Køge, Denmark	Department of Clinical Medicine, Faculty of Health and Medical Sciences, University of Copenhagen, Copenhagen, Denmark
Liam James Elgaard	Quinn	Department of Clinical Immunology, Zealand University Hospital, Køge, Denmark	
Þórunn	Rafnar	deCODE Genetics, Reykjavik, Iceland	
Palle Duun	Rohde	Department of Health Science and Technology, Faculty of Medicine, Aalborg Univeristy, Aalborg, Denmark	
Klaus	Rostgaard	Danish Cancer Society Research Center, Copenhagen, Denmark	Department of Epidemiology Research, Statens Serum Institut, Copenhagen, Denmark
Michael	Schwinn	Department of Clinical Immunology, Copenhagen University Hospital, Rigshospitalet, Copenhagen, Denmark	
Erik	Sørensen	Department of Clinical Immunology, Copenhagen University Hospital, Rigshospitalet, Copenhagen, Denmark	
Kari	Stefansson	deCODE Genetics, Reykjavik, Iceland	
Hreinn	Stefánsson	deCODE Genetics, Reykjavik, Iceland	
Lise Wegner	Thørner	Department of Clinical Immunology, Copenhagen University Hospital, Rigshospitalet, Copenhagen, Denmark	
Unnur	Þorsteinsdóttir	deCODE Genetics, Reykjavik, Iceland	
Mie	Topholm Bruun	Department of Clinical Immunology, Odense University Hospital, Odense, Denmark	
Henrik	Ullum	Statens Serum Institut, Copenhagen, Denmark	

Thomas	Werge	Institute of Biological Psychiatry, Mental Health Centre, Sct. Hans, Copenhagen University Hospital, Roskilde, Denmark	Department of Clinical Medicine, Faculty of Health and Medical Sciences, University of Copenhagen, Copenhagen, Denmark
David	Westergaard	Novo Nordisk Foundation Center for Protein Research, Faculty of Health and Medical Sciences, University of Copenhagen, Copenhagen, Denmark	
