# Supplementary material for: Genome-wide association study reveals a locus in ADARB2 for complete freedom from headache in Danish Blood Donors
Source: Commun Biol. 2024 May 27;7:646. doi: 10.1038/s42003-024-06299-y (PMC11130207; doi:10.1038/s42003-024-06299-y)
Supplement: Supplementary file 3 — Reporting Summary [file 42003_2024_6299_MOESM3_ESM.pdf]

nature portfolio

Corresponding author(s): Thomas Folkmann Hansen

Last updated by author(s): Apr 12, 2024

Reporting Summary

Nature Portfolio wishes to improve the reproducibility of the work that we publish. This form provides structure for consistency and transparency in reporting. For further information on Nature Portfolio policies, see our Editorial Policies and the Editorial Policy Checklist.

Statistics

For all statistical analyses, confirm that the following items are present in the figure legend, table legend, main text, or Methods section.

n/a	Confirmed	
☐	☒	The exact sample size (*n*) for each experimental group/condition, given as a discrete number and unit of measurement
☐	☒	A statement on whether measurements were taken from distinct samples or whether the same sample was measured repeatedly
☐	☒	The statistical test(s) used AND whether they are one- or two-sided<br>*Only common tests should be described solely by name; describe more complex techniques in the Methods section.*
☐	☒	A description of all covariates tested
☐	☒	A description of any assumptions or corrections, such as tests of normality and adjustment for multiple comparisons
☐	☒	A full description of the statistical parameters including central tendency (e.g. means) or other basic estimates (e.g. regression coefficient) AND variation (e.g. standard deviation) or associated estimates of uncertainty (e.g. confidence intervals)
☐	☒	For null hypothesis testing, the test statistic (e.g. *F*, *t*, *r*) with confidence intervals, effect sizes, degrees of freedom and *P* value noted<br>*Give P values as exact values whenever suitable.*
☒	☐	For Bayesian analysis, information on the choice of priors and Markov chain Monte Carlo settings
☒	☐	For hierarchical and complex designs, identification of the appropriate level for tests and full reporting of outcomes
☐	☒	Estimates of effect sizes (e.g. Cohen's *d*, Pearson's *r*), indicating how they were calculated

Our web collection on statistics for biologists contains articles on many of the points above.

Software and code

Policy information about availability of computer code

Data collection	The DBDS is a platform for studies carried out by the Danish blood centers and collaborators. The study is managed by a steering committee who respond to enquiries regarding collaboration. The blood donors participate in the DBDS to increase the scope of their donation, i.e. to help produce valuable research for the benefit of future patients. Additional information can be found on our home page [http://www.dbds.dk]. We invite researchers to collaborate by contacting the steering committee [info@dbds.dk]. Data access requires that projects and applicants obtain permission from the Regional Committees on Health Research Ethics and the Danish Data Protection Agency [http://www.datatilsynet.dk].
Data analysis	All software used in the this study are publicly available. GCTA v1.93 (fastGWAS), PLINK v1.9, FUMA, LDSC, R-package: Tidyverse, ggplot2

For manuscripts utilizing custom algorithms or software that are central to the research but not yet described in published literature, software must be made available to editors and reviewers. We strongly encourage code deposition in a community repository (e.g. GitHub). See the Nature Portfolio guidelines for submitting code & software for further information.

Data

Policy information about availability of data

All manuscripts must include a data availability statement. This statement should provide the following information, where applicable:

- Accession codes, unique identifiers, or web links for publicly available datasets
- A description of any restrictions on data availability
- For clinical datasets or third party data, please ensure that the statement adheres to our policy

The summary statistics are available at Figshare.com with doi: 10.6084/m9.figshare.25575204

Research involving human participants, their data, or biological material

Policy information about studies with human participants or human data. See also policy information about sex, gender (identity/presentation), and sexual orientation and race, ethnicity and racism.

Reporting on sex and gender	We included participants of both sexes. Sex were identified based on genotyping data. We show sex distribution in both cases and controls in the discovery and replication cohorts in Table 1. We adjusted for sex in our genome-wide association analysis.
Reporting on race, ethnicity, or other socially relevant groupings	All included participants were of North European ancestry. We excluded ancestry outliers defined as +/- 5SD from the mean, exluding 219 individuals.
Population characteristics	Co-variates included in the main analysis were sex, age, age squared, the first ten genotype-derived principal components and kinship. In subanalysis we included self-reported questionnaire data on migraine.
Recruitment	Participants were part of the Danish Blood Donor Study (DBDS). The DBDS is a nationwide population-based biobank and research platform that utilizes the existing infrastructure in the Danish blood banks for the prospective cohort. Blood donors must be healthy and comply with strict health criteria to donate blood. Upon enrollment, participants completed a comprehensive questionnaire and a whole blood sample was collected for genotyping. Blood donors are a selected population and in general more healthy than the general population.
Ethics oversight	The DBDS dataset was approved by The Scientific Ethical Committee of Central Denmark (1-10-72-95-13) and of Zealand Region (SJ-740). GWAS studies in DBDS were approved by the National Ethical Committee (NVK-1700407).

Note that full information on the approval of the study protocol must also be provided in the manuscript.

Field-specific reporting

Please select the one below that is the best fit for your research. If you are not sure, read the appropriate sections before making your selection.

☒ Life sciences ☐ Behavioural & social sciences ☐ Ecological, evolutionary & environmental sciences

For a reference copy of the document with all sections, see nature.com/documents/nr-reporting-summary-flat.pdf

Life sciences study design

All studies must disclose on these points even when the disclosure is negative.

Sample size	Sample size was determined by the maximum number of genotyped samples with phenotypic information.
Data exclusions	Samples with missingness >0.03, heterozygosity of +/- 3 standard deviations from the mean and ancestry outliers defined as +/- 5 SD from the mean. SNPs with a missingness >0.02, Hardy Weinberg equilibrium p<1xE-10 and MAF<0.01
Replication	We have been in contact with genetic consortia, and biobanks with genetic data. However, no other group has the same phenotype. We replicated internally in the DBDS.
Randomization	Randomization was not relevant in this cross-sectional case-control study.
Blinding	Blinding was not relevant in this cross-sectional case-control study

Reporting for specific materials, systems and methods

We require information from authors about some types of materials, experimental systems and methods used in many studies. Here, indicate whether each material, system or method listed is relevant to your study. If you are not sure if a list item applies to your research, read the appropriate section before selecting a response.

Materials & experimental systems

n/a	Involved in the study	
☒	☐	Antibodies
☒	☐	Eukaryotic cell lines
☒	☐	Palaeontology and archaeology
☒	☐	Animals and other organisms
☒	☐	Clinical data
☒	☐	Dual use research of concern
☒	☐	Plants

Methods

n/a	Involved in the study	
☒	☐	ChIP-seq
☒	☐	Flow cytometry
☒	☐	MRI-based neuroimaging

Plants

Seed stocks	*Report on the source of all seed stocks or other plant material used. If applicable, state the seed stock centre and catalogue number. If plant specimens were collected from the field, describe the collection location, date and sampling procedures.*
Novel plant genotypes	*Describe the methods by which all novel plant genotypes were produced. This includes those generated by transgenic approaches, gene editing, chemical/radiation-based mutagenesis and hybridization. For transgenic lines, describe the transformation method, the number of independent lines analyzed and the generation upon which experiments were performed. For gene-edited lines, describe the editor used, the endogenous sequence targeted for editing, the targeting guide RNA sequence (if applicable) and how the editor was applied.*
Authentication	*Describe any authentication procedures for each seed stock used or novel genotype generated. Describe any experiments used to assess the effect of a mutation and, where applicable, how potential secondary effects (e.g. second site T-DNA insertions, mosiacism, off-target gene editing) were examined.*
